# Steroid Sparing Effect of 300IR House Dust Mite Sublingual Immunotherapy Tablet in Mite Allergic Rhinitis

**DOI:** 10.1002/clt2.70185

**Published:** 2026-08-11

**Authors:** Oliver Pfaar, Pascal Demoly, Philippe Gevaert, Ludger Klimek, Carmen Vidal, Margitta Worm, Randolf Brehler, Thomas B. Casale, Giorgio Walter Canonica

**Affiliations:** ^1^ Department of Otorhinolaryngology, Head and Neck Surgery Section of Rhinology and Allergy University Hospital Marburg Philipps‐Universität Marburg Marburg Germany; ^2^ University Hospital of Montpellier and IDESP UMR 1318 University of Montpellier – Inserm – Inria Premedical Montpellier France; ^3^ Upper Airways Research Laboratory Department of Otorhinolaryngology Ghent University Ghent Belgium; ^4^ Center for Rhinology and Allergology Wiesbaden Germany; ^5^ Allergy Department Complejo Hospitalario Universitario de Santiago Faculty of Medicine University of Santiago de Compostela Santiago de Compostela Spain; ^6^ Division of Allergy and Immunology Department of Dermatology and Allergology Charité‐Universitätsmedizin Berlin Germany; ^7^ Department of Dermatology Allergology, Occupational Dermatology University Hospital Münster Münster Germany; ^8^ Division of Allergy and Immunology University of South Florida Tampa Florida USA; ^9^ Department of Biomedical Sciences Humanitas University Pieve Emanuele, Milan Italy; ^10^ Personalized Medicine, Asthma and Allergy IRCCS Humanitas Research Hospital Rozzano, Milan Italy

**Keywords:** allergic rhinitis, combined score, house dust mite, steroid sparing, sublingual immunotherapy

## Abstract

**Background:**

In a large randomized controlled trial (NCT02443805) in moderate‐to‐severe house dust mite (HDM) allergic rhinitis (AR) patients with/without controlled asthma, the 300IR HDM sublingual immunotherapy (SLIT) tablet confirmed its efficacy in reducing symptoms and rescue medication (RM) use. This post hoc analysis aimed at assessing the potential steroid sparing effect of this tablet.

**Methods:**

Participants receiving 300IR HDM SLIT‐tablet or placebo for ∼12 months could use antihistamines (AH1), corticosteroids (CS – intranasal (INCS), or oral (OCS)) in a stepwise manner for subjective intolerable nasal/ocular symptoms. The proportions of patients and days with RM intake (overall and by drug class) were assessed over time and compared between groups (Chi‐squared or Wilcoxon rank‐sum tests). An odds that is, ratio of probability of using CS/probability of not using CS was calculated. Mean weekly CS doses were analyzed by ANCOVA.

**Results:**

Of 1262 evaluable patients, ∼60% used at least one CS at baseline: 300IR = 373 and placebo = 396. During treatment, the proportions of patients using RM, oral AH1, and CS and of days with RM use decreased in both groups, systematically in favor of 300IR (*p* < 0.05 at end‐of‐treatment). Odds results indicated SLIT‐patients were more likely not to use CS, whereas placebo‐patients were more likely to use CS (*p* < 0.05 at end‐of‐treatment). After 12 months, 54% SLIT‐patients were able to discontinue CS completely versus 42% placebo‐patients (*p* = 0.0007). Those who were still using CS were taking fewer and lower doses than placebo‐patients.

**Conclusions:**

In moderate to severe HDM‐AR patients, the 300IR HDM SLIT‐tablet showed a steroid sparing effect.

**Abbreviations**
AH1: antihistamines
AIT: allergen immunotherapy
ANCOVA: analysis of covariance
AR: allergic rhinitis
CS: corticosteroids
CSMS: combined symptom and medication score
D far: *Dermatophagoides farinae*
DMS: daily medication score
D pter: *Dermatophagoides pteronyssinus*
EAACI: European Academy of Allergy & Clinical Immunology
FAS: full analysis set
GINA: global initiative for asthma
HDM: house dust mite
INCS: intranasal corticosteroids
IR: index of reactivity
LS: least squares
mFAS: modified full analysis set
MMRM: mixed model with repeated measures
OCS: oral corticosteroids
RCT: randomized controlled trial
RM: rescue medication
RMS: rescue medication score
RTSS: rhinitis total symptom score
SCIT: subcutaneous immunotherapy
SLIT: sublingual immunotherapy
TCRS: total combined rhinitis score
TCS: total combined score
WAO: World Allergy Organization

## 1 Introduction

House dust mites (HDM) constitute one of the main causes of persistent allergic rhinitis (AR) and allergic asthma. The symptom profile in HDM allergy is complex and fluctuates over the year; nasal congestion is particularly bothersome [1, 2, 3, 4]. Inadequately treated moderate‐to‐severe HDM‐AR can significantly disrupt sleep in affected patients, resulting in fatigue and somnolence which negatively impact school/work performance and quality of life, associated with increased health and economic costs [5, 6, 7, 8]. Furthermore, HDM‐AR is a risk factor for the development or worsening of asthma [1, 9, 10].

Given that HDM avoidance measures are generally not effective in everyday life, symptomatic medications are typically used as first‐line treatment for HDM‐AR, and systemic corticosteroids may be considered for very severe clinical situations [9, 10, 11]. However, pharmacotherapy does not modify the natural course of allergic diseases, may be ineffective in some patients and can be associated with bothersome to significant side effects, especially during long‐term use, resulting in substantial additional hidden costs [9, 10, 11, 12, 13, 14, 15, 16].

Allergen immunotherapy (AIT) is the only option that targets the underlying pathophysiology of IgE‐mediated allergy, providing rapid symptomatic relief, long‐lasting effects after discontinuation, and which may prevent the onset or progression of allergic disease [14, 17, 18, 19]. Hence, AIT may be considered in less severe HDM‐AR patients seeking long‐term and preventive benefits, as well as in those with more severe or uncontrolled disease who remain insufficiently managed by symptomatic treatments or wish to avoid their prolonged use and potential adverse effects [14, 20]. Regardless of whether AIT is administered sublingually (SLIT) or subcutaneously (SCIT), the primary treatment goals are decrease in symptom frequency and severity, reduction in symptomatic medication use, and increase in quality of life. In a large phase III randomized controlled trial (RCT), a year‐long treatment with a 300 index of reactivity (IR) SLIT‐tablet formulation of *Dermatophagoides pteronyssinus*/*Dermatophagoides farinae* (D pter/D far) extracts was found to be safe and effective in adults and adolescents with HDM‐AR [21]. Importantly, the treatment effect of the 300IR HDM tablet was found consistent between different compositions of scores combining symptoms and rescue medication use whatever the score (balanced or unbalanced) and the scale size [22, 23, 24].

Focusing on the results of balanced combined scores, strongly recommended by Health Authorities and Scientific Societies (World Allergy Organization [WAO], European Academy of Allergy and Clinical Immunology [EAACI]) as efficacy endpoints [25, 26, 27], the primary objective of this post hoc analysis was to present more detailed data on the patients' medication use during the study. The secondary objective was to determine whether treatment with the 300IR HDM SLIT‐tablet was associated with a steroid‐sparing effect.

## 2 Patients and Methods

### 2.1 Study Design

Methodological details of the phase III RCT conducted in Europe, the United States, Canada, Israel, and Russia between 2015 and 2018 (NCT0244380, EudraCT 2014‐004223‐46) have been previously reported [21]. In brief, eligible patients who gave their written consent/assent to participation entered a 5‐week, single‐blind, placebo‐only run‐in period during which they were asked to record their rhinoconjunctivitis symptoms and medication use daily. Patients with a total combined score (TCS, the sum of rhinitis total symptom score [RTSS] and rescue medication score [RMS]) of at least 5 out of 15 over the last 4 weeks (baseline) were randomized 1:1 (using a computer‐generated list) to receive the 300IR HDM SLIT‐tablet or a placebo tablet for approximately 12 months. Participants were also followed up for 2 weeks after the end of treatment. The study was approved by the local regulatory authorities and appropriate independent ethics committees or institutional review boards and was conducted in accordance with the principles of the Declaration of Helsinki.

### 2.2 Study Population

The study enrolled patients aged 12–65 years with a history of at least one year of clinically documented HDM‐AR with or without asthma (which had to be controlled), a positive skin prick test for D pter and/or D far, and HDM‐specific serum IgE ≥ 3.5 kU/L. The main exclusion criteria were potentially confounding AR, rhinoconjunctivitis or asthma symptoms induced by allergens other than HDMs during the baseline and primary evaluation periods, recent nasal/oral disease or nasal surgery, partly controlled or uncontrolled asthma according to the Global Initiative for Asthma (GINA) definition in force [28], the regular use of asthma medication consistent with GINA treatment steps 3, 4 or 5, a previous life‐threatening asthma attack or exacerbation resulting in admission to an intensive care unit, HDM AIT within the previous 5 years or other AIT within the previous 6 months, and the standard contraindications to AIT [29].

### 2.3 Study Treatments and Allowed Rescue Medications

The 300IR HDM SLIT‐tablet has been described elsewhere [21]. It consisted of a mixture of D pter and D far extracts with a 50/50 total allergenic activity ratio. To maintain blinding, the matching placebo tablet was identical in appearance and taste. Both active and placebo treatments were administered daily for 1 year after an initial 3‐day dose escalation phase.

From the start of the run‐in period to the end of treatment, patients were supplied rescue medications (RM) to manage intolerable nasal/ocular symptoms. They were instructed to use these medications according to a stepwise regimen: (1) oral or topical antihistamine (AH1), (2) intranasal corticosteroid (INCS), and (3) oral corticosteroid (OCS) specifically prescribed by the investigator if intolerable symptoms persisted.

### 2.4 Assessments and Outcomes

The study data were assessed during the 4‐week baseline period, three interim evaluation periods (the 2‐week periods immediately prior to the study visit at months 3, 6 and 9), and the primary evaluation period (4 weeks at the end of the treatment period). Throughout the study, the participants were to record daily (i) the severity (0 = no symptom, 1 = mild, 2 = moderate, 3 = severe) of four individual rhinitis symptoms (nasal pruritus, sneezing, rhinorrhea, and nasal congestion), and (ii) the “strongest” medication used over the previous 24 h (0 = no treatment, 1 = oral AH1, 2 = INCS, 3 = OCS). The daily RTSS, the sum of the rhinitis symptom scores, ranged from 0 to 12. The daily RMS ranged from 0 to 3. Calculated as RTSS + RMS, the average TCS during the 4‐week primary period was the primary efficacy endpoint of this study, ranging from 0 to 15. The combined symptom and medication score (CSMS), a pre‐defined secondary efficacy endpoint, combined the RTSS and the RMS with equal weightings [21]. The CSMS calculated as RTSS/4 + RMS ranged from 0 to 6. Subsequently, based on previously published scores [30], an alternate daily medication score (DMS) was analyzed post hoc imputing a score of four per tablet of oral AH1 (maximum daily score = 4), a score of two per spray of INCS (maximum daily score = 8 i.e., 2 sprays per nostril) and, if applicable, a score of 12 on the day of OCS intake. The DMS ranged from 0 to 12. The total combined rhinitis score (TCRS), equally weighting the RTSS and the DMS, was assessed as complementary post hoc efficacy endpoint. The TCRS calculated as RTSS + DMS ranged from 0 to 24.

The proportions of patients and days with rescue medication (RM) use, overall and by drug class, as well as the mean weekly doses of oral AH1 (number tablets), INCS (number of sprays) were evaluated at each period.

### 2.5 Statistical Analysis

The modified full analysis set (mFAS) comprised all participants who received at least one dose of active treatment or placebo and had at least one assessment of the primary efficacy variable during the primary evaluation period. Analyses were performed in the mFAS for overall population and in the subset of participants who used INCS and/or OCS at least once during the baseline period.

As per the primary efficacy variable, the average balanced combined scores CSMS_0‐6_ and TCRS_0‐24_ and their respective components (RTSS_0‐12_, RMS_0‐3_ and DMS_0‐12_) were analyzed using an analysis of covariance (ANCOVA) on the square root of the average score, with the treatment group as main effect, and the pooled center, square root of the baseline score, age, gender, asthma and sensitization status as covariates; *p*‐values were two‐sided. The absolute between‐group differences (point estimates) were calculated from the back‐transformed least squares (LS) means (i.e., adjusted means taking into account the potential effect of the covariate(s) on the average score). Relative differences (%) equaled 100 × (LS mean 300IR − LS mean placebo)/LS mean placebo). All 95% confidence intervals (CIs) were calculated using the bootstrap method with 10,000 replications.

The proportions of patients and days with RM use (overall, and by drug class) in both the 300IR and placebo groups were analyzed descriptively for each period. Between‐group comparisons were performed using a chi‐squared test or a Wilcoxon rank‐sum test. The evolution of RM use at each evaluation period (Months 3, 6, 9 and 12) versus baseline was assessed using a Mixed Model with Repeated Measures (MMRM), that is, an ANCOVA model with treatment group and evaluation period as main effects, gender, age class, asthma and sensitization status, pooled center and baseline average medication use as covariates, and treatment group x evaluation period as interaction. A MMRM was also used to compare the mean weekly doses of AH1 and CS between both treatment groups at months 3, 6, 9 and 12. In addition, to evaluate the impact of the patients' sensitization or asthma status on the evolution of the CS use (INCS and/or OCS), MMRM analyses with 3‐way interaction (treatment group x evaluation period x sensitization or asthma) were conducted to compare the proportions of days with CS use (INCS and/or OCS) and the mean weekly doses of INCS between both treatment groups at each evaluation period depending on baseline sensitization or asthma status. A longitudinal logistic model including the CS use at baseline in the covariates allowed calculating an odds, that is, ratio of probability of using CS divided by the probability of not using CS at each period in each treatment group.

All statistical analyses were performed with SAS software (version 9.4, SAS Institute Inc., Cary, NC).

## 3 Results

### 3.1 Study Population

A total of 1607 adults and adolescents were randomized and 1601 received at least one dose of treatment (SLIT: 800, placebo: 801) (Supporting Information S1; Figure S1). The mFAS comprised 1262 evaluable patients: 586 in the 300IR group and 676 in the placebo group.

The main demographic and clinical characteristics of the mFAS are presented in Supporting Information S1; Table S1. The mean age was 29.8 years; 551 participants (43.7%) were polysensitized, and 474 (37.6%) had concomitant asthma on inclusion. The mean baseline average scores in the 300IR and placebo groups, respectively, were 2.62 ± 0.912 and 2.53 ± 0.847 for the CSMS_0‐6_, and 10.70 ± 4.227 and 10.33 ± 3.949 for the TCRS_0‐24_. Overall, the baseline characteristics were well balanced, with no notable sociodemographic or disease feature differences between the two treatment groups. At study entry, 1033 (82%) patients were using RM for at least one day, in similar proportions in the two groups, with about two thirds of patients taking oral AH1 and about 60% using any CS for nasal symptoms (Supporting Information S1; Table S2). Less than 7% of patients took OCS and were systematically grouped with those using INCS (subset using CS—INCS and/or OCS).

### 3.2 Balanced Combined Scores

As reported previously [21], during the primary period in the mFAS, the average RTSS_0‐12_ and RMS_0‐3_ were significantly reduced in the 300IR group versus placebo (RTSS_0‐12_ point estimate [95% CI] = −0.64 [−0.96; −0.32], *p* < 0.0001; RMS_0‐3_ point estimate [95% CI] = −0.09 [−0.14; −0.04], *p* = 0.0004; Table 1). The resulting CSMS_0‐6_ was significantly improved in the 300IR group versus placebo (point estimate [95% CI] = −0.26 [−0.38; −0.14], *p* < 0.0001). The alternate medication score, average DMS_0‐12_, also showed a significant reduction in the 300IR group versus placebo (−0.38 [−0.49; −0.26], *p* < 0.0001). Combining RTSS_0‐12_ and DMS_0‐12_, the TCRS_0‐24_ was consistently significantly improved in the 300IR group versus placebo (−1.07 [−1.35; −0.79], *p* < 0.0001). The relative difference from placebo between both balanced combined scores was −18.0% and −18.4%, respectively.

**TABLE 1:** Average scores during the primary evaluation period in the overall population and in patients who used rescue medication during the baseline period.

Average score	Treatment	n	LS mean	Difference in LS mean versus Placebo			Relative LS mean difference	
				Point estimate	[95% CI]	*p*‐value	%	[95% CI]
Overall population								
RTSS	300°IR	586	3.16	−0.64	[−0.96; −0.32]	< 0.0001	−16.8	[−24.2; −8.8]
	Placebo	676	3.79					
RMS	300°IR	586	0.21	−0.09	[−0.14; −0.04]	0.0004	−29.7	[−42.3; −14.8]
	Placebo	676	0.30					
DMS	300°IR	586	0.84	−0.38	[−0.49; −0.26]	< 0.0001	−31.0	[−38.5; −22.6]
	Placebo	676	1.22					
CSMS	300°IR	586	1.19	−0.26	[−0.38; −0.14]	< 0.0001	−18.0	[−25.2; −10.1]
	Placebo	676	1.45					
TCRS	300°IR	586	4.77	−1.07	[−1.35; −0.79]	< 0.0001	−18.4	[−22.7; −13.9]
	Placebo	676	5.85					
Patients with any rescue medication use at baseline								
At least one medication								
CSMS	300°IR	488	1.26	−0.31	[−0.45; −0.17]	< 0.0001	−19.7	[−27.5; −11.4]
	Placebo	545	1.57					
TCRS	300°IR	488	5.06	−1.27	[−1.85; −0.71]	< 0.0001	−20.1	[−28.0; −11.6]
	Placebo	545	6.33					
At least one antihistamine								
CSMS	300°IR	438	1.25	−0.34	[−0.49; −0.19]	< 0.0001	−21.3	[−29.2; −12.5]
	Placebo	489	1.60					
TCRS	300°IR	438	5.07	−1.41	[−2.01; −0.79]	< 0.0001	−21.7	[−29.7; −12.8]
	Placebo	489	6.47					
At least one intranasal and/or oral corticosteroid								
CSMS	300°IR	373	1.37	−0.32	[−0.49; −0.15]	0.0002	−19.0	[−27.6; −9.2]
	Placebo	396	1.70					
TCRS	300°IR	373	5.52	−1.33	[−2.03; −0.62]	0.0002	−19.4	[−28.1; −9.4]
	Placebo	396	6.85					
At least one oral corticosteroid								
CSMS	300°IR	38	1.33	−0.35	[−1.27; 0.51]	0.3128	−20.9	[−57.9; 39.7]
	Placebo	43	1.69					
TCRS	300°IR	38	5.17	−1.34	[−5.05; 1.92]	0.3207	−20.6	[−58.2; 38.4]
	Placebo	43	6.51					

Abbreviations: CI, confidence interval; CSMS, combined symptom and medication score (scale 0–6); DMS, daily medication score (scale 0–12); IR, index of reactivity; LS, least squares; *n*, number of participants in the modified full analysis set with data available for the analysis; RMS, rescue medication score (scale 0–3), RTSS, rhinitis total symptom score (scale 0–12); TCRS, total combined rhinitis score (scale 0–24).

When specifically considering those participants who had taken INCS and/or OCS at least once at baseline, both average CSMS_0‐6_ and TCRS_0‐24_ showed a greater significant difference between 300IR and placebo equal to −0.32 [−0.49; −0.15], and −1.33 [−2.03; −0.62], respectively (*p* = 0.0002), with consistent relative differences (−19.0% and −19.4%; Table 1).

### 3.3 Medication Use in Overall Population

**FIGURE 1:**
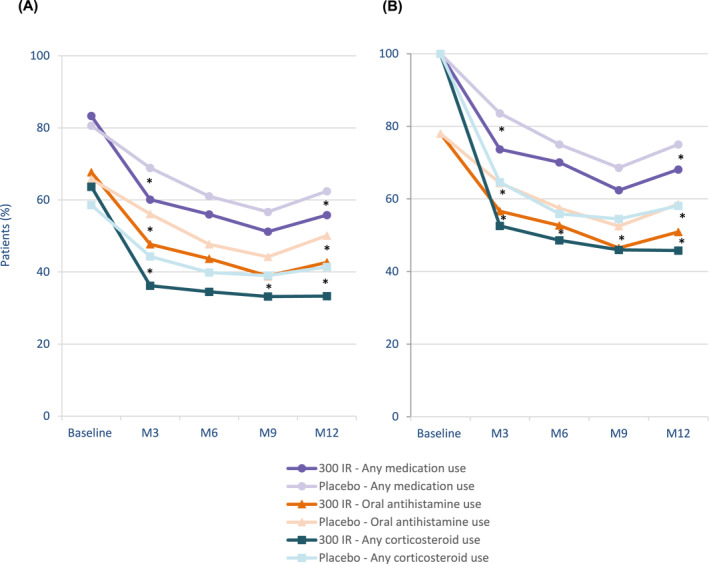
Medication use over time—Proportions of patients in the overall population (mFAS) (A), in the subset of participants having used any corticosteroid at least once during the baseline period (B). *p < 0.05 for 300°IR versus placebo using a Chi‐squared test. IR, index of reactivity; mFAS, modified full analysis set.

**FIGURE 2:**
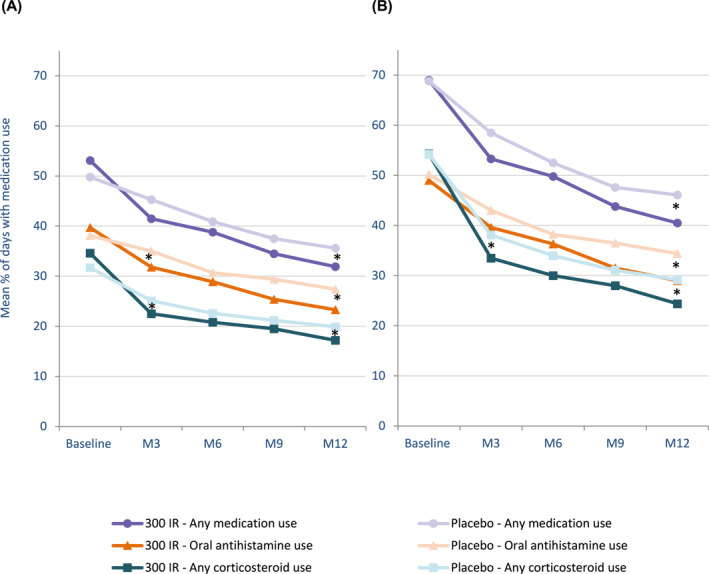
Medication use over time—Proportions of days with use of any medication, oral antihistamine or corticosteroid at each evaluation period in the overall population (mFAS) (A), in the subset of participants having used any corticosteroid at least once during the baseline period (B). *p < 0.05 for 300°IR versus placebo using a Wilcoxon rank‐sum test. IR, index of reactivity; mFAS, modified full analysis set.

During the treatment period, the proportions of patients using RM and more specifically, oral AH1 and CS (INCS and/or OCS) clearly decreased from month 3, systematically in favor of active treatment (Figure 1A and Supporting Information S1; Table S2). In parallel, the proportions of days with RM use, oral AH1 and CS during the evaluation periods progressively decreased over time in both groups (Figure 2A and Supporting Information S1; Table S3). Noteworthy, the change from baseline in the proportion of days with RM use was found significantly different between groups in favor of the 300IR group at all timepoints (except at month 6 for oral AH1 and months 6 and 9 for CS), reaching at the end of treatment −14.9% of days with oral AH1 and ‐16.8% of days with CS for 300IR (placebo: −10.3% and −12.7%, respectively; Supporting Information S1; Figure S2 and Table S4). The mean weekly number of tablets of oral AH1 used by patients decreased over time, from 3.0 tablets to 1.8 in the 300IR group and from 2.9 to 2.2 in the placebo group, corresponding to 40%‐ and 24%‐ relative reductions from baseline, respectively (Table 2). Similarly, the mean weekly number of sprays of INCS fell from 7.8 to 3.3 in the 300IR group and from 6.9 sprays to 4.4 in the placebo group, corresponding to 58%‐ and 36%‐ relative reductions from baseline, respectively. As mentioned above, OCS use was minimal at baseline, averaging 0.13 tablets and 0.08 tablets in the active and placebo groups, respectively. During the study, the 300IR group showed a modest reduction in intake (approximately 0.04 tablets), while no change was observed in the placebo group.

**TABLE 2:** Mean number of doses of oral antihistamine and intranasal corticosteroid used per week at baseline and at each evaluation period in the overall population and in patients having used any corticosteroid at least once during the baseline period.

	300 IR *N* = 586		Placebo *N* = 676		*p*‐value^a^
	*n*	Tablets	*n*	Tablets	
Overall population					
Oral antihistamine					
Baseline	586	3.0 (3.32)	676	2.9 (3.24)	—
M3	577	2.5 [2.2; 2.8]	663	2.8 [2.5; 3.1]	**0.0380**
M6	568	2.2 [2.0; 2.5]	662	2.5 [2.2; 2.8]	0.0918
M9	563	1.9 [1.7; 2.2]	654	2.8 [2.2; 3.7]	**0.0463**
M12 (primary period)	586	1.8 [1.6; 2.1]	676	2.2 [2.0; 2.5]	**0.0091**

	***n***	**Sprays**	***n***	**Sprays**	
Intranasal corticosteroid					
Baseline	586	7.8 (10.64)	676	6.9 (9.35)	—
M3	577	4.4 [3.8; 5.0]	663	5.3 [4.6; 6.0]	**0.0237**
M6	568	4.1 [3.5; 4.8]	662	4.8 [4.2; 5.5]	0.0515
M9	563	3.7 [3.1; 4.4]	654	4.7 [4.1; 5.5]	**0.0098**
M12 (primary period)	586	3.3 [2.8; 4.0]	676	4.4 [3.7; 5.0]	**0.0054**

	**300 IR N = 373**		**Placebo N = 396**		***p*‐value** ^a^
	***n***	**Tablets**	***n***	**Tablets**	
Patients with any corticosteroid use at baseline					
Oral antihistamine					
Baseline	373	3.7 (3.37)	396	3.8 (3.30)	—
M3	369	3.1 [2.8; 3.5]	390	3.4 [3.0; 3.8]	0.2090
M6	364	2.9 [2.5; 3.3]	388	3.1 [2.7; 3.5]	0.4659
M9	359	2.5 [2.1; 2.8]	385	3.0 [2.6; 3.4]	**0.0222**
M12 (primary period)	373	2.4 [2.0; 2.7]	396	2.7 [2.3; 3.0]	0.0839

	***n***	**Sprays**	***n***	**Sprays**	
Intranasal corticosteroid					
Baseline	373	12.2 (11.12)	396	11.7 (9.62)	—
M3	369	6.9 [5.9; 7.9]	390	7.9 [6.8; 9.1]	0.0882
M6	364	6.2 [5.3; 7.2]	388	7.1 [6.0; 8.1]	0.1602
M9	359	5.6 [4.7; 6.7]	385	6.8 [5.7; 7.8]	0.0672
M12 (primary period)	373	5.0 [4.2; 6.0]	396	6.2 [5.2; 7.2]	**0.0405**

*Note:* Weekly doses (i.e., number of tablets or sprays) are presented as descriptive means (SD) at baseline, and as LS means [95% CI] at M3, M6, M9, M12. *p*‐values shown in bold indicate statistical significance.

Abbreviations: IR, index of reactivity; *n*, number of participants in the modified full analysis set with data available for the analysis.

^a^ ANCOVA model with repeated measure on the average weekly number of doses with treatment group and evaluation period as main effects, gender, age class, asthma and sensitization status, pooled center and baseline average weekly number of doses as covariates, and treatment group x evaluation period as interaction.

**FIGURE 3:**
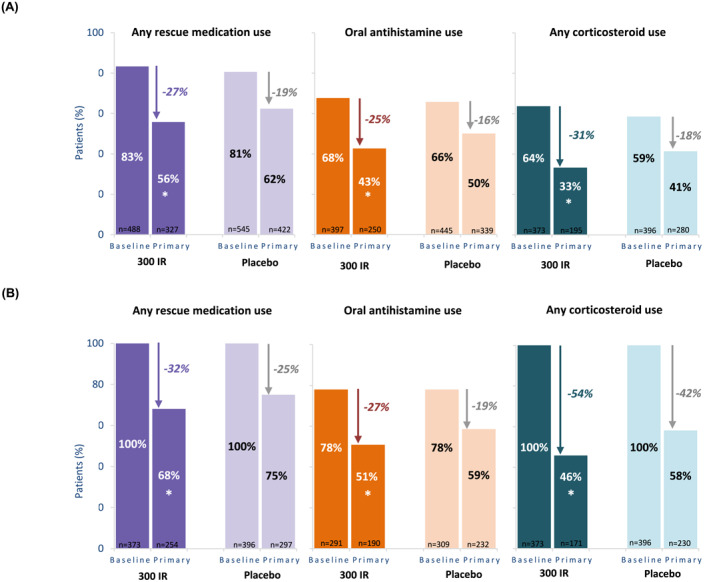
Medication use during baseline and primary evaluation periods (proportions of patients) in the overall population (mFAS) (A), in the subset of participants having used any corticosteroid at least once during the baseline period (B). *p < 0.05 for 300°IR versus placebo using Chi‐squared test. IR, index of reactivity; mFAS, modified full analysis set; n, number of patients at baseline.

At end of treatment (month 12), significantly more patients in the 300IR group than in the placebo group were able to discontinue symptomatic treatments completely: 27% versus 19%, respectively for any RM, 25% versus 16% for oral AH1 and 31% versus 18% for CS (Figure 3A).

The results of MMRM analyses with 3‐way interaction in mono‐ or poly‐sensitized patients or in those with or without asthma at baseline were not statistically significant, whether applied on the proportions of days with CS use (treatment group x evaluation period x sensitization status: *p* = 0.6956; treatment group × evaluation period × asthma: *p* = 0.8567) or on the mean weekly doses of INCS (in sprays, treatment group × evaluation period × sensitization status: *p* = 0.8102; treatment group × evaluation period ×asthma: *p* = 0.7977) (Supporting Information S1; Tables S5 and S6).

### 3.4 Medication Use in Patients Who Used Any Corticosteroid at Least Once at Baseline

**FIGURE 4:**
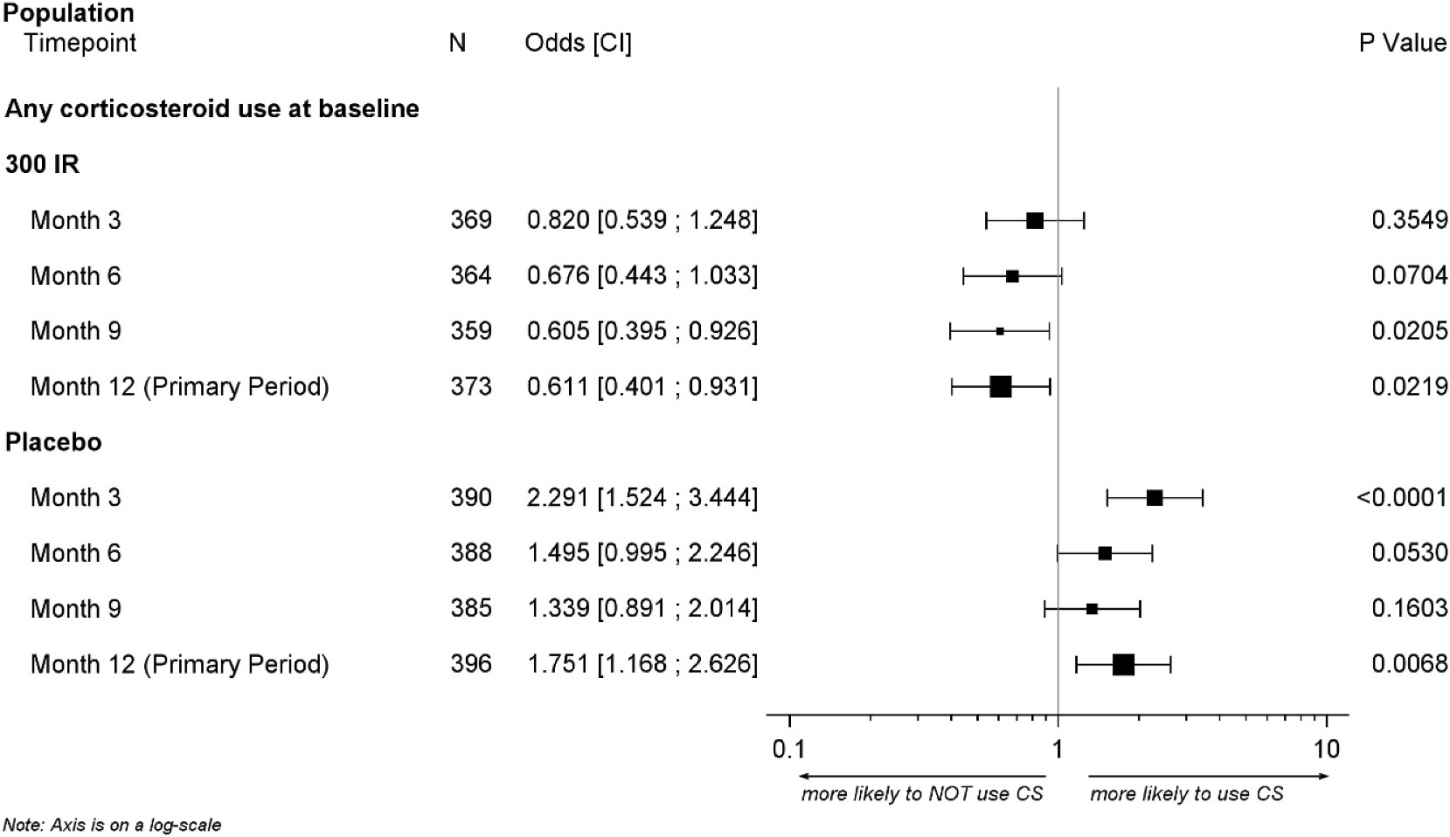
Steroid sparing effect of 300 IR HDM SLIT‐tablet in patients who used a corticosteroid at least once during the baseline period. The odds represent the ratio of probability of using corticosteroids divided by the probability of not using corticosteroids at timepoint in each treatment group. If odds < 1: patients in the treatment group were more likely to NOT use corticosteroids, if odds > 1: patients in the treatment group were more likely to use corticosteroids. HDM, house dust mites; IR, index of reactivity; mFAS, modified full analysis set; *n*, number of participants with data available for the analysis; SLIT, sublingual immunotherapy.

Specifically looking at the subset of participants who had taken INCS and/or OCS at least once during the baseline period (*n* = 769), a continuous decrease in the proportions of patients using RM, oral AH1 and CS and in the proportions of days with medication use was observed during treatment in both groups, with a clear trend in favor of 300IR from month 3 and a significant difference at month 12 (Figures 1B and 2B and Supporting Information S1; Tables S2 and S3). Furthermore, the odds (i.e., the probability ratio of using vs. not using CS) were below 1 in the 300IR group and above 1 in the placebo group at each timepoint from month 3, with significance at the end of treatment in both groups (*p* ≤ 0.02; Figure 4). These results indicate that patients receiving the 300IR HDM SLIT‐tablet were significantly more likely not to use CS than to use them whereas placebo patients were significantly more likely to use CS than not to use them.

By the end of treatment, 54% of patients in the 300IR group were able to discontinue CS completely versus 42% in the placebo group (*p* = 0.0007; Figure 3B). SLIT patients who were still using CS were taking fewer doses than placebo patients. Since OCS were used by 10%–11% patients at baseline and by 3%–5% from month 3 with no significant difference between both groups, these results were mainly driven by INCS with a mean weekly number of sprays falling from 12.2 at baseline to 5.0 at month 12 with the 300IR HDM tablet and from 11.7 to 6.2, respectively, with placebo (Table 2).

## 4 Discussion

As reported previously, the 300IR HDM SLIT‐tablet was confirmed effective in the treatment of adults and adolescents with HDM‐AR with or without asthma, as evidenced by the results (vs. placebo) of symptom and medication scores, whether or not combined, and a quality of life score [21]. The present post hoc analysis specifically focused on two balanced combined scores, the EAACI‐recommended CSMS_0‐6_ pre‐defined as secondary endpoint and the TCRS_0‐24_, a complementary post hoc endpoint. The results showed a significant and consistent reduction in both scores at end of treatment with the 300IR HDM SLIT‐tablet, even greater in the large subset of participants who used an INCS and/or OCS at least once at baseline (relative differences from placebo −19.0%, and −19.4%, respectively). In ANCOVA models of the study primary endpoint conducted by adding interaction between treatment group and sensitization or asthma status at baseline, the interactions did not reach statistical significance, indicating the treatment effect is not impacted by either patients' sensitization status or patients' asthma status (data not shown).

With regards to overall medication use during the study, in the entire population the average RMS was found significantly lower in the 300IR group than in the placebo group over the primary period. Although the proportions of participants taking any medication, and specifically oral AH1 or CS fell over time in both treatment groups, the intergroup differences at end of treatment were significantly in favor of the 300IR HDM SLIT‐tablet. These findings are consistent with the literature data on SLIT formulations for the treatment of intermittent and persistent allergies [30, 31, 32, 33, 34, 35, 36]. Noteworthy, the study population included a somewhat balanced proportion of mono‐sensitized (56%) and poly‐sensitized (44%) patients. The 3‐way interaction analysis showed no difference in the effect of the 300IR HDM SLIT‐tablet on CS use in both subpopulations during and at the end of treatment, suggesting this treatment effect is consistent whether patients are mono‐sensitized or poly‐sensitized.

In contrast to most published phase III trials in patients with pollen allergies, the present study in HDM allergic patients featured a baseline/placebo run‐in period; this enabled the selection of participants who were sufficiently symptomatic to require rescue medication. Nevertheless, medication use during the study was relatively modest (the average RMS during the primary evaluation period equaled 0.30 in the placebo group and 0.21 in the 300 IR group on a 0‐3 scale), in line with the literature data on clinical studies with the 300IR HDM tablet or another HDM SLIT tablet [37, 38, 39, 40]. Noteworthy, the participants in these trials were instructed to take medication only when necessary and in a stepwise manner; this may have prompted them to consume less medication than they would have done in real life. This phenomenon is known as the Hawthorne effect, that is, people's tendency to modifying their behavior when they are aware that they are being monitored. Moreover, the participants were likely to have benefited from optimal care (including guidance on pharmacotherapy) at the various study visits. This guidance and follow‐up might have enabled patients to benefit more from pharmacotherapy than would have been the case for real‐life use of over‐the‐counter pharmacotherapy or alternative treatments. Participation in a closely monitored trial may also explain (at least in part) why medication use also fell in the placebo group [41, 42, 43, 44]. It should be noted that because all participants had access to rescue medication, the observed reduction in the combined scores should not be considered as a pure placebo effect [44]. Even though improvements were noted in the placebo group, the 300IR HDM SLIT‐tablet offered a relevant added clinical benefit. The absolute difference observed between the SLIT and placebo groups may constitute a Minimum Clinically Important Difference (MCID) that would help better interpret the real‐world benefit of the treatment.

In the present study, about 60% of the participants used at least 1 CS (mainly an INCS) during the baseline period – indicating that they had rhinitis symptoms severe enough to trigger such medication intake. The 300IR HDM SLIT‐tablet was found even more effective in these patients than in the overall population, suggesting a greater benefit of AIT in patients experiencing troublesome symptoms and reinforcing its relevance for patients not controlled by CS. As early as 3 months of treatment, more HDM‐AR patients receiving the 300IR HDM tablet than placebo patients could reduce their consumption of RM for a greater number of days. Furthermore, the odds analysis further demonstrated that at each evaluation period, SLIT‐treated patients were more likely not to use CS and placebo patients were more likely to use CS. Nearly half of patients treated with the 300IR HDM SLIT‐tablet no longer required CS at end of treatment. Those who were still using oral AH1 and/or INCS were taking significantly fewer doses than placebo patients. It is important to note that too few patients took OCS during this study, which limited the clear assessment of the effect of the HDM tablet on this drug class. Though a downward trend in OCS intake in the 300 IR group, no significant difference could be observed versus placebo patients due to the small sample size, and the results were mainly driven by the reduction in INCS use.

Altogether, the results confirm the 300IR HDM SLIT‐tablet is associated with a steroid‐sparing effect, offering a valuable clinical benefit for patients who wish to reduce the long‐term use and possible adverse effects of these medications according to AIT indication [14]. Interestingly, the absence of systemic CS use is a key element in the definition of asthma remission, a treatment goal that has been recently adopted in the management of asthma, and including AIT for its potential of disease modification [45, 46, 47]. This concept of remission is also gaining interest in AR. Although a consensus on the definition remains to be found, a proposed definition would include the absence of systemic CS for AR treatment plus minimal or no symptoms and no exacerbations, for at least 1 year [46]. Of note, in previous studies evaluating the long‐term efficacy of HDM SCIT in AR, clinical remission was defined as no longer requiring INCS or oral AH1 [48, 49]. The present results, highlighting the benefits of the 300IR HDM SLIT‐tablet in terms of symptom reduction and nasal/oral steroid sparing, may suggest that this product has the potential to modify the disease and achieve clinical remission in HDM AR.

This study is limited by the post hoc nature of subgroup analyses with modest effect sizes due to limited medication use in the study population. In addition, the short duration of evaluation warrants cautious interpretation. In light of this, the present observation of medication‐sparing effect in patients receiving AIT agrees with real‐world data (i.e., data on AIT products administered during routine patient care). In retrospective longitudinal analyses of prescription or insurance databases in Germany and in France, AIT was consistently safe and associated with long‐term and sustained reductions in AR and asthma medication dispensing, greater than those observed in non‐AIT patients with respiratory allergies (predominantly to HDM, grass or tree pollen) [50, 51, 52, 53, 54, 55]. Although RCTs still constitute the most reliable method for assessing the safety and efficacy of pharmaceutical products, real‐world data analyses can provide further evidence in a more representative population of real life by taking into account the relationships between patient characteristics, lifestyle factors, treatment compliance and regimens, comorbidities, quality of life and clinical outcomes [56, 57]. Moreover, one can expect the placebo effect to be less pronounced in real‐world studies than in clinical trials. On the other hand, it should be acknowledged that causality of medication reductions with AIT is less certain in real‐world studies than in RCTs where patients have free access to rescue medications and are carefully monitored.

As indicated, the original trial included more than a third of patients with concomitant asthma controlled with GINA treatment Step 1 or 2. The 3‐way interaction analysis showed that the asthma status of HDM‐AR patients had no impact on the observed effect of the 300IR HDM SLIT‐tablet on CS savings during and at the end of treatment. However, since asthma control and inhaled CS use were not primary endpoints of this trial, further studies are warranted to confirm the steroid sparing effect of this tablet on asthma. Furthermore, the potential relationship between the reduction in steroid dose and immunological parameters, as well as the relationship between changes in spirometry parameters and immunological parameters, would be interesting to observe over time.

In conclusion, this post hoc analysis of a large, multinational RCT confirmed that the 300IR HDM SLIT‐tablet not only significantly reduced symptoms, but also enabled HDM‐AR patients to achieve a key therapeutic goal of reducing long‐term use of pharmacotherapy, with positive odds of no longer requiring corticosteroids. The observed decrease in medication use (particularly INCS) over time, in terms of proportions of days and doses, adds to the data accumulated in this context supporting the value of AIT, including in severe AR patients with or without comorbid asthma.

## Author Contributions

**Oliver Pfaar:** investigation, writing – review and editing. **Pascal Demoly:** conceptualization, investigation, writing – review and editing, methodology. **Philippe Gevaert:** investigation, writing – review and editing. **Ludger Klimek:** investigation, writing – review and editing. **Carmen Vidal:** investigation, writing – review and editing. **Margitta Worm:** investigation, writing – review and editing. **Randolf Brehler:** writing – review and editing. **Thomas B. Casale:** conceptualization, investigation, methodology, writing – review and editing. **Giorgio Walter Canonica:** writing – review and editing.

## Funding

The present study was sponsored and funded by Stallergenes Greer, Antony, France, which also provided funding for the open access publication of this article.

## Conflicts of Interest

Oliver Pfaar reports grants and/or personal fees from Stallergenes Greer during the clinical trial reported, and he reports grants and/or personal fees and/or travel support from AEDA, Alfried Krupp Krankenhaus, ALK‐Abelló, Allergopharma, Almirall, Altamira Therapeutics, ASIT Biotech, AstraZeneca, Bencard Allergie GmbH/Allergy Therapeutics, Biologixpharma, Blueprint, Breazy Health, Cliantha, Deutsche AllergieLiga e.V., Cogitando GmbH/medcram, Deutsche Forschungsgemeinschaft, Dustri‐Verlag, ECM Expro&Conference Management GmBH, Forum für Medizinische Fortbildung, Georg‐Thieme‐Verlag, GSK, HAL Allergy Holding B.V./HAL Allergie GmbH, Inmunotek, Ingress Health, Institut für Disease Management, IQVIA Commercial, Japanese Society of Allergology, Königlich Dänisches Generalkonsulat, Laboratorios LETI/LETI Pharma, Lilly, Lofarma, Medcram/Cogitando, Medizinische Hochschule Hannover, med update europe GmbH, Meinhardt Congress GmbH, Novartis, Paul‐Ehrlich‐Institut, Paul‐Martini‐Stiftung, PneumoLive, Pohl‐Boskamp, Procter & Gamble, Red Maple Trials Inc., Regeneron, RG Aerztefortbildung, ROXALL Medizin, Sanofi Aventis, Sanofi Genzyme, Springer Publisher, Stallergenes Greer, streamedup! GmbH, Technical University Dresden, John Wiley & sons publishers, Wort & Bild Verlag, Verlag ME, Gemeinnütziger Verein zur Förderung der Allergiefortbildung (VFAF), all outside the submitted work. Oliver Pfaar is board member of ENT section of the European Academy of Allergy and Clinical Immunology (EAACI) and member the external board of directors of the German Society of Allergy and Clinical Immunology (DGAKI); coordinator, main‐ or co‐author of different position papers and guidelines in rhinology, allergology and allergen‐immunotherapy; and he is Editor‐in‐Chief of Clinical Translational Allergy. Pascal Demoly has no direct financial interests; he has received institutional grants for teaching, research and humanitarian activities from: ALK‐Abelló, AstraZeneca, Chiesi, GlaxoSmithKline, Menarini, Puressentiel, Stallergenes Greer, ThermoFisher Scientific, Viatris, and Zambon. Philippe Gevaert reports grants, consulting fees and/or payment or honoraria for lectures, presentations, speakers bureaus, manuscript writing or educational events from Eli Lilly, GlaxoSmithKline, Sanofi‐Regeneron and Stallergenes Greer, not related to the article submitted; and support for attending meetings and/or travel from Stallergenes Greer. Ludger Klimek reports grants or contracts, and/or consulting fees, and/or payment for expert testimony from ALK‐Abelló, Allergopharma, Allergy Therapeutics, AstraZeneca, GlaxoSmithKline, HAL Allergie, Immunotek, Leti Pharma, Novartis, Regeneron Pharmaceuticals, Sanofi, Stallergenes Greer, Viatris, paid to him or to his institution; and other financial or non‐financial interest from AeDA, DGHNO, Deutsche Gesellschaft für Allergologie und klinische Immunologie, HNO‐BV, GPA, EAACI. Carmen Vidal has no conflicts of interest to declare. Margitta Worm reports consulting fees and/or payment or honoraria from AbbVie Deutschland GmbH & Co. KG, Aimmune Therapeutics UK Limited, ALK‐Abelló Arzneimittel GmbH, Allergopharma GmbH & Co KG, Almirall Hermal GmbH, Amgen GmbH, AstraZeneca GmbH, Bayer AG, Bencard Allergy GmbH, Bioprojet Pharma, Boehringer Ingelheim Pharma GmbH &Co.KG, Bristol Myers Squibb GmbH & Co. KGaA, Galderma Laboratorium GmbH, Glaxo Smith Kline GmbH & Co. KG, Infectopharm Arzneimittel und Consilium GmbH, LEO Pharma GmbH, Lilly Deutschland GmbH, Mylan Germany GmbH (A Viatris Company), Novartis AG, Octapharm AG, Pfizer Pharma GmbH, Sanofi‐Aventis Deutschland GmbH/Genzyme Europe B. B. and Stallergenes GmbH. Randolf Brehler reports honoraria for lectures, speakers fees, or consulting fees from Aeda, ALK‐Abelló, Allergopharma, Apothekerkammer Westfalen‐Lippe, AstraZeneca, Behring, Bioprojet, BVDD, Coglitando, DGP, DIATER, ECM Expo&Conference Management GmbH, Forum für med. Fortbildung FomF GmbH, GlaxoSmithKline, HAL, Janssen‐Cilag GmBH, Leti, Lilly, MedUpdate, Medscape, Mein Allergie Portal, Novartis, RG‐Gesellschaft für Information und Organisation mbH, Sanofi, Stallergenes, Takeda, Thermo‐Fischer, med update GmbH. Thomas B. Casale reports grants to his institution from ALK‐Abelló and Stallergenes Greer, and consulting fees from ThermoFisher Scientific. Giorgio Walter Canonica reports having received consulting fees, and/or payment or honoraria for lectures, presentations, speakers bureaus, manuscript writing or educational events (to him or his institution) from AstraZeneca, Celltrion, Chiesi, Faes, Farmaceutici, Firma, Genentech, GlaxoSmithKline, GrandPharma, Guidotti, Innovacaremd, Menarini, Novartis, OmPharma, Opella, RedMaple, Sanofi Aventis, Sanofi Genzyme, Stallergenes Greer and Uriach Pharma.

## Supporting information


Supporting Information S1

## Data Availability

The data that supports the findings of this study are available in the supplementary material of this article.
