# Supplementary material for: Steroid Sparing Effect of 300IR House Dust Mite Sublingual Immunotherapy Tablet in Mite Allergic Rhinitis
Source: Clin Transl Allergy. 2026 Aug 11;16(8):e70185. doi: 10.1002/clt2.70185 (PMC13460716; doi:10.1002/clt2.70185)
Supplement: Supplementary file 1 [file CLT2-16-e70185-s001.docx]

**Steroid sparing effect of 300IR house dust mite sublingual immunotherapy tablet in mite allergic rhinitis**

Oliver Pfaar, Pascal Demoly, Philippe Gevaert, Ludger Klimek, Carmen Vidal, Margitta Worm, Randolf Brehler, Thomas B. Casale, and Giorgio Walter Canonica

**SUPPORTING INFORMATION**

**Figure S1.** Patient disposition

FAS, full analysis set; IR, index of reactivity; mFAS, modified full analysis set; SS, Safety set

Assessed for eligibility (n=4,267)

Excluded (n=2,660)

□ Not meeting inclusion criteria (n=2,513)

□ Declined to participate (n=113)

□ Other reasons (n=34)

Analyzed (mFAS, n=586 of 802, 73.1%)
□ Excluded from analysis, due to exclusion from the SS or a lack of data for the primary efficacy evaluation during the primary period (n=216)

Discontinued intervention (n=213 of 802, 26.6%)

□ Withdrawn due to adverse events (n=100)

□ Withdrew consent (n=73)

□ Lost to follow-up (n=19)

□ Other reasons (n=21)

Allocated to 300°IR (n=802)

□ Received allocated intervention (n=799)

□ Did not receive allocated intervention (n=3)

*(note: SS=800)*

Discontinued intervention (n=127 of 805, 15.8%)

□ Withdrawn due to adverse events (n=18)

□ Withdrew consent (n=74)

□ Lost to follow-up (n=14)

□ Other reasons (n=21)

Allocated to placebo (n=805)

□ Received allocated intervention (n=802)

□ Did not receive allocated intervention (n=3)

*(note: SS=801)*

Analyzed (mFAS, n=676 of 805, 84.0%)
□ Excluded from analysis, due to exclusion from the SS or a lack of data for the primary efficacy evaluation during the primary period (n=129)

Randomized 1:1 (n=1,607)

(343 adolescents and 1,264 adults)

Analyzed (FAS, n=765 of 805, 95.0%)
□ Excluded from analysis, due to exclusion from the SS or a lack of data for the primary efficacy evaluation during the treatment period (n=40)

Analyzed (FAS, n=711 of 802, 88.7%)
□ Excluded from analysis, due to exclusion from the SS or a lack of data for the primary efficacy evaluation during the treatment period (n=91)

**Figure S2.** Change from baseline in the proportions of days with use of any medication, oral antihistamine or corticosteroid at each evaluation period using a Mixed Model with Repeated Measures in the overall population (mFAS). *p<0.05 for 300°IR vs. placebo.

IR, index of reactivity; mFAS, modified full analysis set

Mean % of days by period

**Table S1.** Main baseline characteristics in the mFAS

|  | **300 IR N=586** | **Placebo N=676** | **Overall N=1,262** |
| --- | --- | --- | --- |
| **Gender** [n (%)] |  |  |  |
| Male | 301 (51.4) | 327(48.4) | 628 (49.8) |
| Female | 285 (48.6) | 349 (51.6) | 634 (50.2) |
| **Age (**years) |  |  |  |
| Mean (SD) | 29.9 (13.3) | 29.6 (12.7) | 29.8 (13.0) |
| Median | 28.0 | 29.0 | 28.0 |
| Range | 12-66 | 12-64 | 12-66 |
| **Age classes** [n (%)] |  |  |  |
| Adults | 455 (77.6) | 532 (78.7) | 987 (78.2) |
| Adolescents | 131 (22.4) | 144 (21.3) | 275 (21.8) |
| **Geographical region** [n (%)] |  |  |  |
| North America (USA & Canada) | 164 (28.0) | 203 (30.0) | 367 (29.1) |
| Europe, Israel, Russia | 422 (72.0) | 473 (70.0) | 895 (70.9) |
| **Race** [n (%)] |  |  |  |
| White | 549 (93.7) | 623 (92.2) | 1,172 (92.9) |
| Black or African American | 19 (3.2) | 28 (4.1) | 47 (3.7) |
| American Indian or Alaska Native | 0 | 1 (0.1) | 1 (0.1) |
| Asian | 15 (2.6) | 19 (2.8) | 34 (2.7) |
| Native Hawaiian or Other Pacific Islander | 2 (0.3) | 0 | 2 (0.2) |
| Multiple | 1 (0.2) | 2 (0.3) | 3 (0.2) |
| Unknown | 0 | 3 (0.4) | 3 (0.2) |
| **Duration of HDM-induced allergic rhinitis** (years) |  |  |  |
| Mean (SD) | 11.3 (10.55) | 11.3 (9.76) | 11.3 (10.13) |
| Median | 7.3 | 8.4 | 8.0 |
| Range | 0.8−58.9 | 0.4−50.2 | 0.4−58.9 |
| **Baseline sensitization status** [n (%)] |  |  |  |
| Monosensitized | 338 (57.7) | 373 (55.2) | 711 (56.3) |
| Polysensitized | 248 (42.3) | 303 (44.8) | 551 (43.7) |
| **Concomitant asthma** [n (%)] |  |  |  |
| Presence | 227 (38.7) | 247 (36.5) | 474 (37.6) |
| Absence | 359 (61.3) | 429 (63.5) | 788 (62.4) |
| **Average TCS at baseline** |  |  |  |
| Mean (SD) | 7.87 (2.124) | 7.68 (1.940) | 7.77 (2.029) |
| 95%CI | [7.70; 8.05] | [7.54; 7.83] | [7.66; 7.88] |
| Median | 7.51 | 7.36 | 7.42 |
| Range | 3.8−14.4 | 4.4−14.0 | 3.8−14.4 |
| **Average CSMS at baseline** |  |  |  |
| Mean (SD) | 2.62 (0.912) | 2.53 (0.847) | 2.57 (0.879) |
| 95%CI | [2.54; 2.69] | [2.46; 2.59] | [2.52; 2.62] |
| Median | 2.47 | 2.38 | 2.41 |
| Range | 1.2−5.8 | 1.1−5.5 | 1.1−5.8 |
| **Average TCRS at baseline** |  |  |  |
| Mean (SD) | 10.70 (4.227) | 10.33 (3.949) | 10.50 (4.083) |
| 95%CI | [10.36; 11.04] | [10.03; 10.62] | [10.27; 10.72] |
| Median | 9.67 | 9.42 | 9.47 |
| Range | 4.7−24.0 | 4.4−24.0 | 4.4−24.0 |

Abbreviations: CI: confidence interval; CSMS, combined symptom and medication score (scale 0-6); IR, index of reactivity; mFAS, modified full analysis set; SD, standard deviation; mFAS, modified full analysis set; N, number of participants in the modified full analysis set; n, number of participants with data; TCRS, total combined rhinitis score (scale 0-24); TCS, total combined score (scale 0-15).

**Table S2.** Proportions of participants with at least one day of medication use at baseline and at each evaluation period in the overall population (mFAS) and in the subset of participants having used any corticosteroid at least once during the baseline period

| **Overall population** | | | | | |
| --- | --- | --- | --- | --- | --- |
|  | **300 IR N=586** | **Placebo N=676** | | **p-value**^†^ | |
| *Any medication [n(%)]* | | | | | |
| **Baseline** | 488 (83.3) | | 545 (80.6) | | 0.2222 |
| **M3** | 347 (60.1) | | 457 (68.9) | | **0.0012** |
| **M6** | 318 (56.0) | | 404 (61.0) | | 0.0734 |
| **M9** | 288 (51.2) | | 371 (56.7) | | 0.0517 |
| **M12 (Primary Period)** | 327 (55.8) | | 422 (62.4) | | **0.0169** |
| *Oral antihistamine [n(%)]* | | | | | |
| **Baseline** | 397 (67.7) | | 445 (65.8) | | 0.4706 |
| **M3** | 275 (47.7) | | 372 (56.1) | | **0.0030** |
| **M6** | 248 (43.7) | | 316 (47.7) | | 0.1530 |
| **M9** | 219 (38.9) | | 289 (44.2) | | 0.0620 |
| **M12 (Primary Period)** | 250 (42.7) | | 339 (50.1) | | **0.0079** |
| *Any corticosteroid [n(%)]* | | | | | |
| **Baseline** | 373 (63.7) | | 396 (58.6) | | 0.0655 |
| **M3** | 209 (36.2) | | 294 (44.3) | | **0.0037** |
| **M6** | 196 (34.5) | | 264 (39.9) | | 0.0522 |
| **M9** | 187 (33.2) | | 255 (39.0) | | **0.0367** |
| **M12 (Primary Period)** | 195 (33.3) | | 280 (41.4) | | **0.0029** |
| *Intranasal corticosteroid [n(%)]* | | | | | |
| **Baseline** | 367 (62.6) | | 389 (57.5) | | 0.0661 |
| **M3** | 207 (35.9) | | 289 (43.6) | | **0.0057** |
| **M6** | 195 (34.3) | | 260 (39.3) | | 0.0734 |
| **M9** | 183 (32.5) | | 252 (38.5) | | **0.0287** |
| **M12 (Primary Period)** | 189 (32.3) | | 276 (40.8) | | **0.0016** |
| *Oral corticosteroid [n(%)]* | | | | | |
| **Baseline** | 38 (6.5) | | 43 (6.4) | | 0.9287 |
| **M3** | 10 (1.7) | | 23 (3.5) | | 0.0582 |
| **M6** | 14 (2.5) | | 18 (2.7) | | 0.7800 |
| **M9** | 13 (2.3) | | 21 (3.2) | | 0.3411 |
| **M12 (Primary Period)** | 14 (2.4) | | 23 (3.4) | | 0.2872 |

| **Patients with any corticosteroid use at baseline** | | | | | |
| --- | --- | --- | --- | --- | --- |
|  | **300 IR N=373** | **Placebo N=396** | | **p-value**^†^ | |
| *Any medication [n(%)]* | | | | | |
| **Baseline** | 373 (100) | | 396 (100) | | - |
| **M3** | 272 (73.7) | | 326 (83.6) | | **0.0009** |
| **M6** | 255 (70.1) | | 291 (75.0) | | 0.1286 |
| **M9** | 224 (62.4) | | 264 (68.6) | | 0.0764 |
| **M12 (Primary Period)** | 254 (68.1) | | 297 (75.0) | | **0.0338** |
| *Oral antihistamine [n(%)]* | | | | | |
| **Baseline** | 291 (78.0) | | 309 (78.0) | | 0.9962 |
| **M3** | 209 (56.6) | | 251 (64.4) | | **0.0296** |
| **M6** | 192 (52.7) | | 223 (57.5) | | 0.1927 |
| **M9** | 167 (46.5) | | 202 (52.5) | | 0.1048 |
| **M12 (Primary Period)** | 190 (50.9) | | 232 (58.6) | | **0.0332** |
| *Any corticosteroid [n(%)]* | | | | | |
| **Baseline** | 373 (100) | | 396 (100) | | - |
| **M3** | 194 (52.6) | | 252 (64.6) | | **0.0008** |
| **M6** | 177 (48.6) | | 217 (55.9) | | **0.0451** |
| **M9** | 165 (46.0) | | 210 (54.5) | | **0.0193** |
| **M12 (Primary Period)** | 171 (45.8) | | 230 (58.1) | | **0.0007** |
| *Intranasal corticosteroid [n(%)]* | | | | | |
| **Baseline** | 367 (98.4) | | 389 (98.2) | | 0.8642 |
| **M3** | 192 (52.0) | | 249 (63.8) | | **0.0010** |
| **M6** | 177 (48.6) | | 213 (54.9) | | 0.0855 |
| **M9** | 162 (45.1) | | 208 (54.0) | | **0.0153** |
| **M12 (Primary Period)** | 168 (45.0) | | 227 (57.3) | | **0.0007** |
| *Oral corticosteroid [n(%)]* | | | | | |
| **Baseline** | 38 (10.2) | | 43 (10.9) | | 0.7620 |
| **M3** | 10 (2.7) | | 20 (5.1) | | 0.0875 |
| **M6** | 13 (3.6) | | 15 (3.9) | | 0.8312 |
| **M9** | 12 (3.3) | | 19 (4.9) | | 0.2774 |
| **M12 (Primary Period)** | 11 (2.9) | | 17 (4.3) | | 0.3201 |

^†^ Chi-squared test

Abbreviations: CI, confidence interval; IR, index of reactivity; mFAS, modified full analysis set; N, number of participants in the modified full analysis set; n, number of participants with data available for the analysis.

**Table S3.** Proportions of days with at least one medication use at baseline and during each evaluation period, in the overall population (mFAS) and in the subset of participants having used any corticosteroid at least once during the baseline period

| **Overall population** | | | | | |
| --- | --- | --- | --- | --- | --- |
|  | **300 IR N=586** | **Placebo N=676** | | **p-value**^†^ | |
| *Any medication [mean % days (SD)]* | | | | | |
| **Baseline** | 53.1 (40.88) | | 49.8 (40.72) | | - |
| **M3** | 41.5 (43.68) | | 45.3 (42.23) | | 0.0601 |
| **M6** | 38.8 (43.49) | | 40.9 (42.58) | | 0.2464 |
| **M9** | 34.5 (42.30) | | 37.5 (42.61) | | 0.1231 |
| **M12 (Primary Period)** | 31.9 (40.97) | | 35.6 (40.66) | | **0.0250** |
| *Oral antihistamine [mean % days (SD)]* | | | | | |
| **Baseline** | 39.7 (41.67) | | 38.1 (40.94) | | - |
| **M3** | 31.8 (41.76) | | 35.0 (41.57) | | **0.0374** |
| **M6** | 28.9 (40.92) | | 30.7 (40.53) | | 0.2639 |
| **M9** | 25.4 (38.85) | | 29.4 (41.22) | | 0.0547 |
| **M12 (Primary Period)** | 23.3 (37.76) | | 27.4 (39.05) | | **0.0096** |
| *Any corticosteroid [mean % days (SD)]* | | | | | |
| **Baseline** | 34.6 (39.72) | | 31.7 (38.68) | | - |
| **M3** | 22.5 (36.94) | | 25.1 (36.62) | | **0.0207** |
| **M6** | 20.8 (35.90) | | 22.6 (36.05) | | 0.1153 |
| **M9** | 19.5 (35.12) | | 21.2 (34.32) | | 0.0831 |
| **M12 (Primary Period)** | 17.2 (33.11) | | 19.9 (33.00) | | **0.0067** |
| *Intranasal corticosteroid [mean % days (SD)]* | | | | | |
| **Baseline** | 34.1 (39.65) | | 31.5 (38.66) | | - |
| **M3** | 22.1 (36.67) | | 24.6 (36.34) | | **0.0264** |
| **M6** | 20.6 (35.80) | | 22.5 (36.01) | | 0.1315 |
| **M9** | 19.0 (34.76) | | 21.0 (34.23) | | 0.0638 |
| **M12 (Primary Period)** | 16.7 (32.90) | | 19.7 (33.02) | | **0.0037** |
| *Oral corticosteroid [mean % days (SD)]* | | | | | |
| **Baseline** | 1.1 (7.95) | | 1.0 (7.28) | | - |
| **M3** | 0.6 (6.59) | | 1.0 (7.90) | | 0.0599 |
| **M6** | 0.5 (4.41) | | 0.7 (6.16) | | 0.7784 |
| **M9** | 0.8 (7.17) | | 1.0 (7.37) | | 0.3356 |
| **M12 (Primary Period)** | 0.6 (5.36) | | 0.7 (6.13) | | 0.2937 |

| **Patients with any corticosteroid use at baseline** | | | | | | |
| --- | --- | --- | --- | --- | --- | --- |
|  | **300 IR N=373** | **Placebo N=396** | | **p-value**^†^ | | |
| *Any medication [mean % days (SD)]* | | | | | | |
| **Baseline** | 69.9 (34.13) | | 68.8 (33.59) | | - |  |
| **M3** | 53.3 (43.22) | | 58.5 (40.18) | | 0.1042 |  |
| **M6** | 49.9 (43.44) | | 52.5 (42.67) | | 0.3123 |  |
| **M9** | 43.8 (43.59) | | 47.6 (43.36) | | 0.1837 |  |
| **M12 (Primary Period)** | 40.5 (42.53) | | 46.1 (41.54) | | **0.0292** |  |
| *Oral antihistamine [mean % days (SD)]* | | | | | | |
| **Baseline** | 49.0 (41.94) | | 50.2 (41.53) | | - |  |
| **M3** | 39.6 (43.65) | | 43.0 (42.97) | | 0.1509 |  |
| **M6** | 36.3 (43.09) | | 38.2 (42.45) | | 0.3969 |  |
| **M9** | 31.5 (41.36) | | 36.5 (43.59) | | 0.0822 |  |
| **M12 (Primary Period)** | 29.0 (40.35) | | 34.4 (41.72) | | **0.0277** |  |
| *Any corticosteroid [mean % days (SD)]* | | | | | | |
| **Baseline** | 54.3 (37.52) | | 54.2 (36.56) | | - |  |
| **M3** | 33.5 (41.16) | | 38.1 (40.11) | | **0.0181** |  |
| **M6** | 30.0 (40.16) | | 34.0 (40.53) | | 0.0946 |  |
| **M9** | 28.0 (39.34) | | 31.1 (38.57) | | 0.0766 |  |
| **M12 (Primary Period)** | 24.4 (37.50) | | 29.2 (36.99) | | **0.0032** |  |
| *Intranasal corticosteroid [mean % days (SD)]* | | | | | | |
| **Baseline** | 53.6 (37.77) | | 53.8 (36.80) | | - |  |
| **M3** | 32.8 (40.95) | | 37.4 (39.91) | | **0.0197** |  |
| **M6** | 29.8 (40.13) | | 33.8 (40.55) | | 0.1240 |  |
| **M9** | 27.4 (39.19) | | 30.7 (38.52) | | 0.0646 |  |
| **M12 (Primary Period)** | 24.0 (37.52) | | 28.9 (37.07) | | **0.0026** |  |
| *Oral corticosteroid [mean % days (SD)]* | | | | | | |
| **Baseline** | 1.8 (9.91) | | 1.8 (9.45) | | - |  |
| **M3** | 1.0 (8.22) | | 1.5 (9.87) | | 0.0916 |  |
| **M6** | 0.6 (3.97) | | 1.0 (7.88) | | 0.8198 |  |
| **M9** | 0.9 (7.27) | | 1.6 (9.35) | | 0.2677 |  |
| **M12 (Primary Period)** | 0.5 (4.19) | | 0.8 (6.10) | | 0.3254 |  |

The evaluation periods consisted of the two weeks immediately prior to the study visit at months 3, 6 and 9, and the four weeks during the baseline run-in period and at the end of the treatment period (primary period).

^†^ Wilcoxon rank-sum test

Abbreviations: CI, confidence interval; IR, index of reactivity; mFAS, modified full analysis set; N, number of participants in the modified full analysis set

**Table S4.** Change from baseline in the proportions of days with use of any medication, oral antihistamine or corticosteroid at each evaluation period, in the overall population (mFAS)

| **Overall population** | | | | | |
| --- | --- | --- | --- | --- | --- |
|  | **300 IR N=586** | **Placebo N=676** | **Difference in LS mean *vs.* Placebo** | | |
|  | LS Mean [95% CI] ^†^ | LS Mean [95% CI] ^†^ | Point estimate [95% CI] | | p-value ^‡^ |
| *Any medication* | | | | | |
| **M3** | -10.8 [-13.71; -7.87] | -4.7 [-7.35; -1.81] | -6.1 [-9.46; -2.69] | **0.0006** | |
| **M6** | -13.6 [-16.51; -10.69] | -9.6 [-12.40; -6.68] | -4.0 [-7.61; -0.48] | **0.0335** | |
| **M9** | -17.8 [-20.80; -14.39] | -12.8 [-15.83; -9.86] | -4.9 [-8.23; -1.18] | **0.0149** | |
| **M12 (Primary Period)** | -20.0 [-23.16; -16.74] | -14.4 [-17.55; -11.41] | -5.6 [-9.12; -1.66] | **0.0038** | |
| *Oral antihistamine* | | | | | |
| **M3** | -6.7 [-9.44; -3.74] | -2.6 [-5.04; 0.30] | -4.1 [-7.49; -0.42] | **0.0197** | |
| **M6** | -9.6 [-12.51; -6.55] | -7.2 [-10.18; -4.35] | -2.4 [-5.89; 1.03] | 0.2001 | |
| **M9** | -13.2 [-16.09; -10.07] | -8.4 [-11.55; -5.56] | -4.8 [-8.27; -1.04] | **0.0127** | |
| **M12 (Primary Period)** | -14.9 [-17.94; -11.74] | -10.3 [-13.25; -7.58] | -4.6 [-7.98; -1.00] | **0.0105** | |
| *Any corticosteroid* | | | | | |
| **M3** | -11.6 [-14.02; -8.95] | -7.5 [-10.07; -4.78] | -4.1 [-7.27; -0.87] | **0.0105** | |
| **M6** | -13.4 [-16.02; -10.54] | -10.1 [-12.97; -7.46] | -3.2 [-6.20; 0.07] | 0.0520 | |
| **M9** | -14.7 [-17.49; -11.83] | -11.6 [-14.38; -8.89] | -3.1 [-6.20; 0.30] | 0.0619 | |
| **M12 (Primary Period)** | -16.8 [-19.62; -13.94] | -12.7 [-15.58; -9.95] | -4.1 [-7.23; -1.00] | **0.0107** | |

^†^ LS Mean percentage of days by period

^‡^ Mixed Model with Repeated Measures

Abbreviations: CI, confidence interval; IR, index of reactivity; mFAS, modified full analysis set; N, number of participants in the modified full analysis set

**Table S5.** Impact of the patients' sensitization status on the evolution of corticosteroid use over time: Three-way interaction analyses on the proportion of days with at least one corticosteroid use and on mean weekly doses of intranasal corticosteroids, at each evaluation period in the overall population (mFAS)

| **Proportion of days with at least one corticosteroid use** | | | | | | |
| --- | --- | --- | --- | --- | --- | --- |
| **Model Effect** | | | | | | **p-value** ^‡^ |
| Treatment group | | | | | | 0.0093 |
| Evaluation period | | | | | | <0.0001 |
| Gender | | | | | | 0.0616 |
| Age class | | | | | | 0.9600 |
| Sensitization status | | | | | | 0.4762 |
| Asthma status | | | | | | 0.8068 |
| Pooled center | | | | | | 0.0298 |
| Baseline average | | | | | | <0.0001 |
| Evaluation period * Sensitization status at baseline | | | | | | 0.5256 |
| Treatment group x Evaluation period | | | | | | 0.7718 |
| Treatment group x Sensitization status at baseline | | | | | | 0.5794 |
| Treatment group x Evaluation period x Sensitization status at baseline | | | | | | 0.6956 |
| **Mono-sensitized patients** | | | | | | |
|  | 300 IR N=338 | | | Placebo N=373 | | p-value |
|  | n | LS Mean [95% CI] ^†^ | n | | LS Mean [95% CI] ^†^ |  |
| **M3** | 334 | 21.9 [18.05; 25.58] | 369 | | 25.6 [22.12; 29.16] | 0.0819 |
| **M6** | 330 | 19.3 [15.93; 23.21] | 368 | | 23.3 [19.84; 26.87] | 0.0735 |
| **M9** | 324 | 17.3 [13.93; 21.10] | 364 | | 21.6 [17.99; 25.00] | 0.0529 |
| **M12 (Primary Period)** | 338 | 14.9 [11.41; 18.49] | 373 | | 20.1 [16.83; 23.52] | 0.0152 |
| **Polysensitized patients** | | | | | | |
|  | 300 IR N=248 | | | Placebo N=303 | | p-value |
|  | n | LS Mean [95% CI] ^†^ | n | | LS Mean [95% CI] ^†^ |  |
| **M3** | 243 | 21.3 [17.81; 25.12] | 294 | | 26.0 [22.17; 29.86] | 0.0550 |
| **M6** | 238 | 20.6 [17.07; 24.26] | 294 | | 22.9 [19.14; 26.55] | 0.3606 |
| **M9** | 239 | 20.3 [16.61; 23.99] | 290 | | 21.8 [18.04; 25.39] | 0.5455 |
| **M12 (Primary Period)** | 248 | 18.5 [14.89; 22.17] | 303 | | 21.1 [17.41; 25.08] | 0.2857 |

| **Weekly number of doses (in sprays) of intranasal corticosteroids** | | | | | | |
| --- | --- | --- | --- | --- | --- | --- |
| **Model Effect** | | | | | **p-value** ^‡^ | |
| Treatment group | | | | | 0.0052 | |
| Evaluation period | | | | | <0.0001 | |
| Gender | | | | | 0.0210 | |
| Age class | | | | | 0.8919 | |
| Sensitization status | | | | | 0.4654 | |
| Asthma status | | | | | 0.8132 | |
| Pooled center | | | | | 0.0027 | |
| Baseline average | | | | | <0.0001 | |
| Evaluation period * Sensitization status at baseline | | | | | 0.4548 | |
| Treatment group x Evaluation period | | | | | 0.9301 | |
| Treatment group x Sensitization status at baseline | | | | | 0.5899 | |
| Treatment group x Evaluation period x Sensitization status at baseline | | | | | 0.8102 | |
| **Mono-sensitized patients** | | | | | | |
|  | 300 IR N=338 | | Placebo N=373 | | | p-value |
|  | n | LS Mean [95% CI] | n | LS Mean [95% CI] | |  |
| **M3** | 334 | 4.4 [3.49; 5.22] | 369 | 5.3 [4.41; 6.16] | | 0.0802 |
| **M6** | 330 | 4.0 [3.15; 4.88] | 368 | 4.8 [4.01; 5.72] | | 0.1194 |
| **M9** | 324 | 3.3 [2.57; 4.15] | 364 | 4.7 [3.74; 5.55] | | 0.0131 |
| **M12 (Primary Period)** | 338 | 3.0 [2.27; 3.81] | 373 | 4.3 [3.51; 5.08] | | 0.0100 |
| **Polysensitized patients** | | | | | | |
|  | 300 IR N=248 | | Placebo N=303 | | | p-value |
|  | n | LS Mean [95% CI] | n | LS Mean [95% CI] | |  |
| **M3** | 243 | 4.4 [3.63; 5.33] | 294 | 5.3 [4.36; 6.29] | | 0.1473 |
| **M6** | 238 | 4.1 [3.29; 4.93] | 294 | 4.8 [3.94; 5.70] | | 0.2310 |
| **M9** | 239 | 4.2 [3.32; 5.08] | 290 | 4.8 [3.91; 5.79] | | 0.2910 |
| **M12 (Primary Period)** | 248 | 3.7 [2.91; 4.57] | 303 | 4.5 [3.55; 5.38] | | 0.2094 |

^†^ LS Mean percentage of days by period

^‡^ Mixed Model with Repeated Measures

Abbreviations: CI, confidence interval; IR, index of reactivity; mFAS, modified full analysis set; N, number of participants in the modified full analysis set; n, number of participants with data available for the analysis.

**Table S6.** Impact of the patients' asthma status on the evolution of corticosteroid use over time: Three-way interaction analyses on the proportion of days with at least one corticosteroid use and on mean weekly doses of intranasal corticosteroids, at each evaluation period in the overall population (mFAS)

| **Proportion of days with at least one corticosteroid use** | | | | | | |
| --- | --- | --- | --- | --- | --- | --- |
| **Model Effect** | | | | | | **p-value** ^‡^ |
| Treatment group | | | | | | 0.0097 |
| Evaluation period | | | | | | <0.0001 |
| Gender | | | | | | 0.0631 |
| Age class | | | | | | 0.9489 |
| Sensitization status | | | | | | 0.5320 |
| Asthma status | | | | | | 0.7487 |
| Pooled center | | | | | | 0.0301 |
| Baseline average | | | | | | <0.0001 |
| Evaluation period * Asthma at baseline | | | | | | 0.8948 |
| Treatment group x Evaluation period | | | | | | 0.7252 |
| Treatment group x Asthma at baseline | | | | | | 0.9121 |
| Treatment group x Evaluation period x Asthma at baseline | | | | | | 0.8567 |
| **Asthma = Yes** | | | | | | |
|  | 300 IR N=227 | | | Placebo N=247 | | p-value |
|  | n | LS Mean [95% CI] ^†^ | n | | LS Mean [95% CI] ^†^ |  |
| **M3** | 221 | 21.4 [17.90; 25.27] | 241 | | 26.4 [22.32; 30.40] | 0.0573 |
| **M6** | 216 | 19.7 [16.03; 23.64] | 243 | | 22.3 [18.24; 26.42] | 0.3408 |
| **M9** | 219 | 18.6 [14.53; 22.96] | 239 | | 21.0 [16.94; 25.01] | 0.3841 |
| **M12 (Primary Period)** | 227 | 16.4 [12.28; 20.26] | 247 | | 20.3 [16.24; 24.23] | 0.1445 |
| **Asthma = No** | | | | | | |
|  | 300 IR N=359 | | | Placebo N=429 | | p-value |
|  | n | LS Mean [95% CI] ^†^ | n | | LS Mean [95% CI] ^†^ |  |
| **M3** | 356 | 22.0 [18.43; 25.50] | 422 | | 25.5 [22.24; 28.70] | 0.0773 |
| **M6** | 352 | 20.1 [16.86; 23.92] | 419 | | 23.7 [20.49; 27.08] | 0.0884 |
| **M9** | 344 | 18.7 [15.47; 22.41] | 415 | | 22.2 [19.06; 25.69] | 0.0940 |
| **M12 (Primary Period)** | 359 | 16.6 [13.20; 20.15] | 429 | | 20.9 [17.83; 23.94] | 0.0369 |

| **Weekly number of doses (in sprays) of intranasal corticosteroids** | | | | | | |
| --- | --- | --- | --- | --- | --- | --- |
| **Model Effect** | | | | | **p-value** ^‡^ | |
| Treatment group | | | | | 0.0051 | |
| Evaluation period | | | | | <0.0001 | |
| Gender | | | | | 0.0217 | |
| Age class | | | | | 0.8831 | |
| Sensitization status | | | | | 0.5346 | |
| Asthma status | | | | | 0.6727 | |
| Pooled center | | | | | 0.0028 | |
| Baseline average | | | | | <0.0001 | |
| Evaluation period * Asthma at baseline | | | | | 0.5129 | |
| Treatment group x Evaluation period | | | | | 0.9138 | |
| Treatment group x Asthma at baseline | | | | | 0.9921 | |
| Treatment group x Evaluation period x Asthma at baseline | | | | | 0.7977 | |
| **Asthma = Yes** | | | | | | |
|  | 300 IR N=227 | | Placebo N=247 | | | p-value |
|  | n | LS Mean [95% CI] | n | LS Mean [95% CI] | |  |
| **M3** | 221 | 4.4 [3.57; 5.34] | 241 | 5.4 [4.43; 6.26] | | 0.1480 |
| **M6** | 216 | 3.7 [2.92; 4.64] | 243 | 4.6 [3.73; 5.54] | | 0.1742 |
| **M9** | 219 | 3.7 [2.77; 4.64] | 239 | 4.5 [3.58; 5.43] | | 0.2231 |
| **M12 (Primary Period)** | 227 | 3.3 [2.38; 4.23] | 247 | 4.4 [3.50; 5.31] | | 0.0602 |
| **Asthma = No** | | | | | | |
|  | 300 IR N=359 | | Placebo N=429 | | | p-value |
|  | n | LS Mean [95% CI] | n | LS Mean [95% CI] | |  |
| **M3** | 356 | 4.4 [3.60; 5.30] | 422 | 5.3 [4.48; 6.10] | | 0.0821 |
| **M6** | 352 | 4.3 [3.46; 5.17] | 419 | 5.0 [4.19; 5.81] | | 0.1647 |
| **M9** | 344 | 3.7 [2.95; 4.52] | 415 | 4.9 [4.04; 5.78] | | 0.0211 |
| **M12 (Primary Period)** | 359 | 3.4 [2.64; 4.13] | 429 | 4.4 [3.62; 5.05] | | 0.0394 |

^†^ LS Mean percentage of days by period

^‡^ Mixed Model with Repeated Measures

Abbreviations: CI, confidence interval; IR, index of reactivity; mFAS, modified full analysis set; N, number of participants in the modified full analysis set; n, number of participants with data available for the analysis.
